# Physicomechanical Properties of Tissue Conditioner Reinforced with Glass Fibers

**DOI:** 10.3390/bioengineering12050515

**Published:** 2025-05-13

**Authors:** Aftab Ahmed Khan, Abdulaziz Abdullah AlKhureif, Eraj Humayun Mirza, Raghad Khalid AlHassoun, Aisha Wasi, Jukka Matinlinna

**Affiliations:** 1 Dental Biomaterials Research Chair, College of Applied Medical Sciences, King Saud University, Riyadh 11451, Saudi Arabia; aalkhuraif@ksu.edu.sa (A.A.A.); raghadalhassoun11@gmail.com (R.K.A.); aishawasi98@hotmail.com (A.W.); 2 Biomedical Engineering Department, NED University of Engineering and Technology, Karachi 69230, Pakistan; dreraj@neduet.edu.pk; 3 Biomaterials Science, Division of Dentistry, University of Manchester, Manchester 13M 9PL, UK; jukka.matinlinna@manchester.ac.uk

**Keywords:** tissue conditioner, glass fiber, aging, physicomechanical properties, viscogel

## Abstract

Tissue conditioners are temporary lining materials applied to dentures to soothe and cushion inflamed or traumatized oral tissues, typically resulting from ill-fitting dentures. This laboratory study aimed to evaluate the physicomechanical properties of a clinical tissue conditioner with 0.5 and 1 wt.% of silanized, micron-sized, E-glass fibers. The experimental tissue conditioners were characterized based on their molecular structure, surface roughness, contact angle, tensile strength, dimensional stability, water sorption, and solubility. The results were analyzed by two-way ANOVA (factors: material composition and aging) and the post hoc Tukey’s test. FTIR analysis revealed characteristic peaks at 1710–1720 cm^−1^, 2800–3000 cm^−1^, and 1400 cm^−1^, indicating a strong interaction between the tissue conditioner and the micron-sized glass fibers. Tensile strength was highest at baseline but declined in all groups after 14 days of aging, with the 0.5 wt.% glass fiber group showing the least reduction. Linear dimensional changes remained consistent across all groups. Surface roughness increased in all groups after 14 days, though the 0.5 wt.% glass fiber group exhibited the smallest increase. Water contact angles ranged from 71° to 92°, suggesting adequate surface wettability for clinical use. The experimental groups consistently demonstrated lower water sorption and solubility values. The 0.5 wt.% glass fiber formulation showed the potential to improve clinical performance by its reduced water sorption and solubility. However, long-term studies and clinical trials are necessary to validate the clinical effectiveness of this formulation.

## 1. Introduction

A full-arch, removable acrylic denture is the most popular and economically viable option for edentulous arches [1]. A removable denture enhances a person’s quality of life by improving oral functions such as speaking, chewing, and appearance. However, prolonged wear of dentures or inadequate denture cleanliness can cause oral infections, particularly in elderly patients who are immunocompromised or handicapped [2]. The most common oral condition among denture wearers is denture-induced stomatitis, which affects around 65% of patients; fungal infections caused by *Candida albicans* account for 93% of cases [3,4]. This problem can be treated by replacing the old denture, medication, improving diet, and using denture soft liners [5].

Temporary soft denture liners, commonly referred to as tissue conditioners, have been used since the 1960s to facilitate the healing of compromised oral mucosa [6]. They reduce the masticatory forces and prevent direct pressure application to the mucosa and alveolar ridge while offering a cushioning effect beneath the ill-fitting denture [7,8]. Tissue conditioner consists of a powder of poly(ethyl methacrylate) and a liquid component containing plasticizers and ethanol that combine to form a soft, gel-like material [9]. Tissue conditioners are recommended for short-term use (from a few days to a maximum of 4 weeks) to promote the healing and recovery of denture-supporting tissues. The ideal characteristics of a tissue conditioner include color and dimensional stability, biocompatibility, ease of manipulation and processing, good bond strength, resilience, and low hardness and water sorption [10]. However, over time, tissue conditioners develop surface roughness, colonize microbes, leach plasticizers, discolor, undergo reduced adhesion to the denture base, and decline in their intended cushioning effect [5,9,11]. The reduction in bond strength and the low resilience of tissue conditioners contribute to their mechanical weakness [10,11].

Recent advancements in tissue conditioners have focused on integrating antimicrobial agents such as silver nanoparticles (up to 0.5%), conventional antifungals (e.g., nystatin), non-organic additives (e.g., silver zeolite), and herbal extracts (e.g., tea tree oil) to combat pathogens like *Candida albicans*, *Staphylococcus aureus*, and *Streptococcus mutans* [3,11,12]. However, existing studies have not extensively explored methods to significantly improve the physicomechanical attributes of tissue conditioners. Glass fibers are composed of a strong and complex three-dimensional network of silicon and oxygen atoms, making them highly suitable for reinforcing dental materials [13]. Glass fibers are also esthetically pleasing and biocompatible [14]. Silanization of glass fibers is essential [15] to promote chemical bonding between the fibers and the polymer matrix in the composite [16,17,18].

This laboratory study aimed to enhance the properties of tissue conditioners through the incorporation of micron-sized glass fibers. To the authors’ knowledge, no studies have ever reported the use of glass fibers or any other fibers in tissue conditioners. It was hypothesized that the addition of micron-sized glass fibers would enhance the physicomechanical properties of tissue conditioner and that these properties in glass fiber-modified tissue conditioner would remain largely unaffected after 24 h and 14 days of water aging.

## 2. Materials and Methods

### 2.1. Synthesis and Fabrication of Experimental Tissue Conditioner Samples

Visco-Gel™ (Dentsply Sirona, Bensheim, Germany), a widely used tissue conditioner, was chosen for this study. Its powder composition was blended with micron-sized E-glass fibers, measuring 150 µm in length, 16 µm in diameter, and having an aspect ratio of 11:1. These fibers typically consist of SiO_2_ (52–56 wt.%), Al_2_O_3_ (12–16 wt.%), CaO (16–25 wt.%), and B_2_O_3_ (8–13 wt.%). The glass fibers were obtained pre-silanized from Sigma-Aldrich (St. Louis, MO, USA) with 3-[trimethoxysilyl]propyl methacrylate and then blended into the powder component of the tissue conditioner at two weight ratios (0.5 and 1.0 wt.%). The mixing was performed with an amalgamator (Promix^TM^; Dentsply Caulk, York, PA, USA) for 20 s. After mixing, samples were prepared using a standard amount of 2.2 g of powder to 1.8 g of Visco-Gel™ liquid, mixing for 30 s in a rubber bowl. The mixture was then placed into a cylindrical-shaped mold (6 mm in diameter and 3 mm in height) to fabricate as many samples as needed, by repeating this procedure for surface roughness, Fourier transform infrared spectroscopy, water contact angle, water sorption, and solubility testing. The same procedure was repeated with fresh material to prepare the remaining samples. Disc-shaped samples with a 30 mm diameter and 2.5 mm thickness were fabricated separately for the linear dimensional stability test. Dumbbell-shaped samples (20 mm in length and a neck diameter of 6 mm) were fabricated for tensile strength testing.

After an initial setting time of 10 min, all samples were removed from the molds and stored in distilled water at 37 °C. Sixteen samples were prepared per group, with eight samples tested after 24 h and the remaining eight tested after 14 days of water aging at 37 °C.

### 2.2. Fourier Transform Infrared Spectroscopy

The molecular structure and functional group of the silanized glass fibers were analyzed and identified using a NICOLET iS5 spectrometer (Thermo Scientific, Waltham, MA, USA). The analysis was performed using a monolithic diamond attenuated total reflectance (ATR iD7). The sample was positioned on the ATR-FTIR accessory crystal, and the spectrum was recorded in the range of 4000 to 500 cm^−1^ with a resolution of 2 cm^−1^.

### 2.3. Surface Roughness Test

The prepared samples were analyzed with a 3D optical non-contact surface profilometer (ContourGT, Bruker, Campbell, CA, USA) using white light interferometry to measure the mean surface roughness (S_a_, µm). A 5× magnification objective lens was used to focus on the required area for scanning. The accuracy of surface roughness measurements was controlled using Vision64 (v 5.30) application software. The surface roughness was measured both after 24 h and after 14 days of water aging. Eight samples per group were evaluated.

### 2.4. Contact Angle Evaluation

A Tensiometer (Theta Lite, Dyne Technology, Staffordshire, UK) was used to record the contact angle measurements of the samples. The test to measure the contact angle of the tissue conditioner was performed after 24 h and after 14 days of water aging. Eight samples per group were tested. The contact angle of the samples was measured by calculating the angle formed by placing a 3 µL droplet of deionized water on the sample surface [19].

### 2.5. Tensile Strength Test

**Figure 1:**
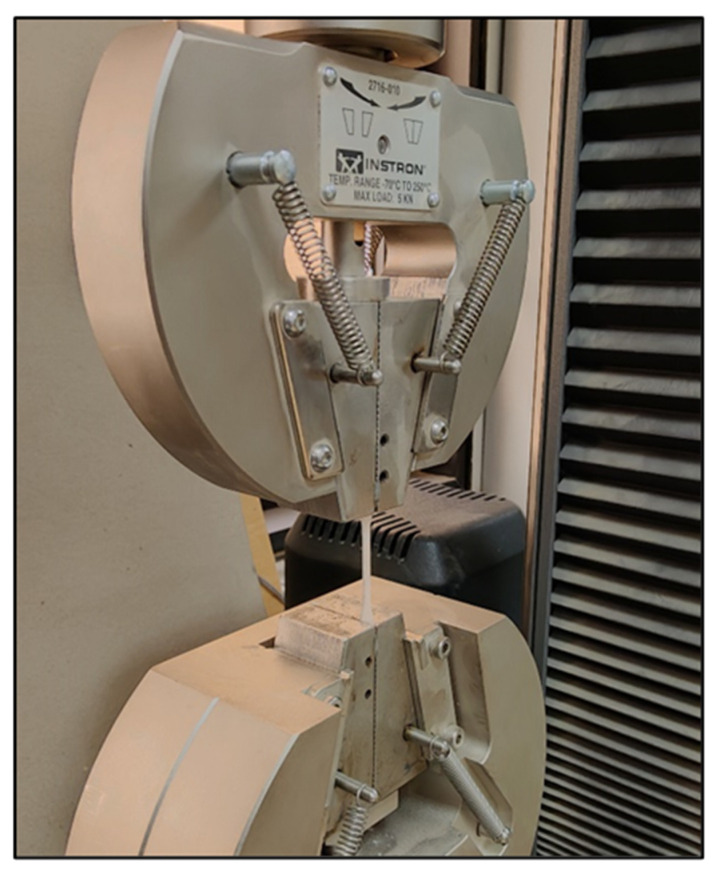
An experimental tissue conditioner sample under the tensile load.

A universal testing machine (Model no. 3369 Instron, Canton, MA, USA) equipped with a 5 kN load cell was used to perform tensile strength testing of the samples with a crosshead speed of 2 mm/min, as shown in Figure 1. The dumbbell-shaped samples (20 mm in length with a neck diameter of 6 mm; *n* = 8/group) were evaluated. The ends of the sample were secured to the grips of a tensile device using cyanoacrylate (Super Glue, Henkel/Loctite, Westlake, CA, USA). The tensile strength was calculated using proprietary software (Bluehill ver.3.15) integrated with the universal testing machine, based on the standard formula:

Tensile strength = F/A

where F = maximum load at failure and A = cross-sectional area of the sample.

### 2.6. Linear Dimensional Stability Test

**Figure 2:**
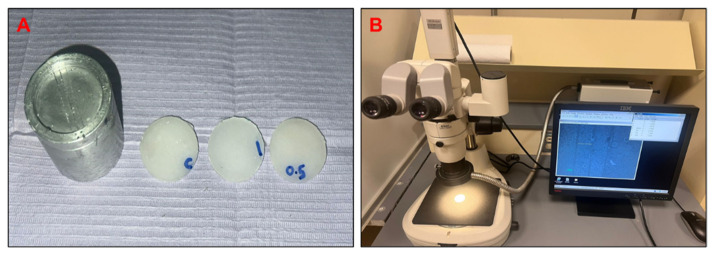
(**A**) Calibrated mold used for the preparation of disk-shaped samples for the linear dimensional stability test and (**B**) stereomicroscopic evaluation of reference lines.

In accordance with ISO standard 10139-2: 2016 [20], dimensional stability testing was conducted; however, disc-shaped samples (30 mm in diameter; 2.5 mm thick) were prepared using a calibrated mold, which differs from the rectangular specimen dimensions specified in the standard. A precisely calibrated aluminum block mold was used, featuring lines 50 μm in width, spaced 24.805 mm apart (L1). Five samples were made for each group. The samples were taken out from the mold after 20 min and stored in distilled water at 37 °C with each subgroup of five samples immersed in 200 mL of distilled water. After 24 h, a light stereomicroscope (Nikon SM2-10, Tokyo, Japan) at a magnification of 20× fitted with a measuring tool was used to determine the distance between the two reference lines on the flat face of the samples (L_2_). Similarly, aging group samples were stored separately under the same conditions (Figure 2).

The linear dimension changes were calculated using the following equation, as per the ISO standard 10139-1:2018 [21] explained by Chladek et al. [22].

Ref. [23],

$$Dimensional\;change=\frac{L_{1}-L_{2}}{L_{1}}\times 100$$

### 2.7. Water Sorption and Solubility Tests

For water solubility and sorption tests, five disc-shaped samples from each subgroup were fabricated. After 1 h, the samples were removed from the mold and placed in a desiccator until a constant weight (±0.5 mg) was measured, designated as the initial weight of the sample (M_1_). Next, the samples were placed in covered plastic jars, filled with 100 mL of distilled water, and stored at 37 °C. After 24 h, the samples were removed, gently blotted dry, and weighed (M_2_), followed by drying in a desiccator until a constant mass was achieved (M_3_). The water sorption and solubility were calculated using the following equations [24]:

Water sorption (%) = (M_2_ − M_3_)/M_1_ × 100

Water solubility (%) = (M_1_ − M_3_)/M_1_ × 100

The same procedure was applied to two separate groups of samples stored in distilled water for 14 days. The water (100 mL per sample) was not replaced throughout the storage period. The 24-h and 14-day measurements were conducted on independent sets of samples and were not cumulative.

### 2.8. Statistical Analysis

The data were statistically analyzed using SPSS software version 28 (IBM, New York, NY, USA). The descriptive data were expressed in mean and standard deviation. A two-way ANOVA was performed to test for significant differences between the measuring intervals. A *p*-value of <0.05 was considered statistically significant. Tukey’s post hoc tests were performed following the ANOVA analysis to distinguish the significant differences within and between the groups.

## 3. Results

### 3.1. FTIR

**Figure 3:**
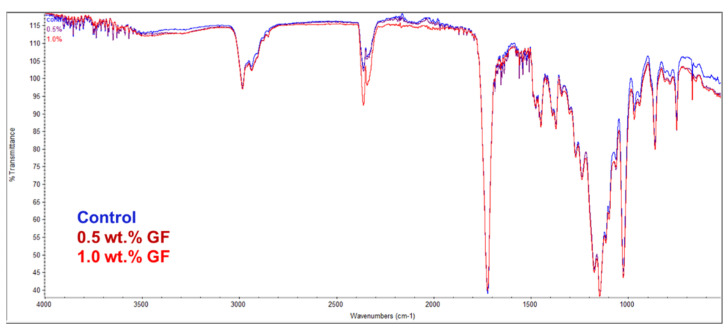
FTIR spectra of tissue conditioners modified with glass fibers (0.5 wt.% and 1.0 wt.%) and unmodified tissue conditioners (control).

The FTIR spectra of the study groups are displayed in Figure 3. Similar spectral patterns were observed among the control group and the glass fiber-reinforced groups, indicating potential interactions between the tissue conditioner matrix and the incorporated glass fibers. The carbonyl group (C=O bond) of the poly(ethyl methacrylate) was observed at 1720 cm^−1^. The peaks at 1710–1720 cm^−1^ corresponded to the ester group in poly(ethyl methacrylate). The polymer bond of C–H was represented by the peak between 2800–3000 cm^−1^ and 1400 cm^−1^. The variations in the glass fiber peaks, observed between 1000 and 1200 cm^−1^, indicated the silica of the glass fibers and may have been caused by changes in the concentration of glass fibers in the matrix. The dibutyl phthalate’s C-O peaks were observed between 1160 and 1173 cm^−1^ [21,22].

### 3.2. Tensile Strength

**Figure 4:**
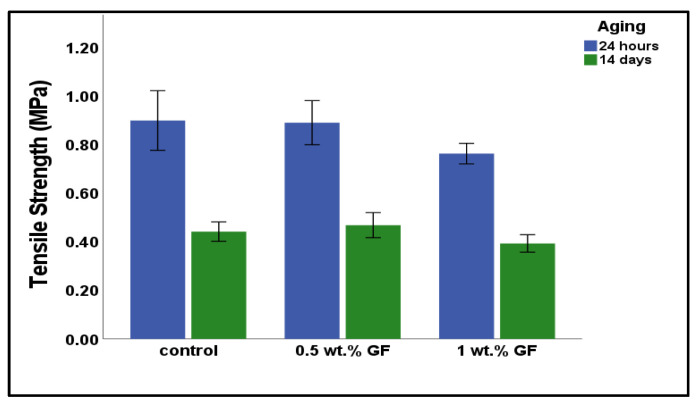
Mean tensile strength of tested materials at 24 h and 14 days. The term ‘GF’ denotes glass fiber.

The tensile strengths of the control and experimental groups after 24 h and 14 days of aging are shown in Figure 4. At 24 h, the control group had the highest tensile strength, followed by the 0.5 wt.% glass fiber group and the 1 wt.% glass fiber group. At the end of day 14, the 1 wt.% glass fiber group showed the lowest strength, followed by the control group, while the 0.5 wt.% glass fiber group exhibited the highest tensile strength. However, the differences among the groups were not statistically significant at either time point (*p* = 0.102 after 24 h and *p* = 0.080 after 14 days).

### 3.3. Linear Dimensional Stability

**Figure 5:**
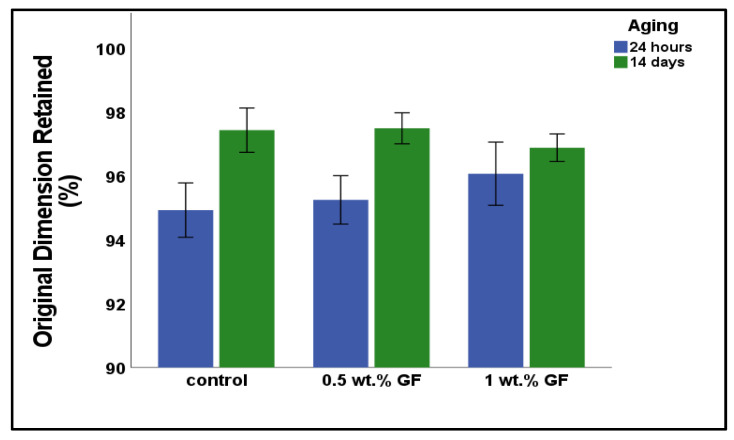
Original dimension retained (in %) of the tested materials at 24 h and 14 days.

Figure 5 shows the % of the original linear dimension retained for each study group. After 24 h, the control group exhibited the greatest shrinkage, retaining 94.932 ± 0.952% of its original dimension. This was followed by the 0.5 wt.% GF group (95.252 ± 0.849%), and the 1 wt.% GF group (96.072 ± 1.107%). However, the differences in shrinkage among the groups were not statistically significant (*p* = 0.205). After 14 days, all groups exhibited an increase in their linear dimensions over the 24 h values but were still smaller than at the baseline. The dimensional change at 14 days was 97.5 ± 0.5, 96.9 ± 0.5, and 97.4 ± 0.8 for the 0.5% GF, 1% GF, and control groups, respectively. Again, the differences among the groups after 14 days were non-significant (*p* = 0.263).

Table 1 presents the mean and standard deviation values for the surface roughness, water sorption, water solubility, and water contact angle of the study groups after 24 h and 14 days of aging. All groups exhibited increased surface roughness, water sorption, and solubility after 14 days compared to 24 h of evaluation, with the control group showing the highest values for deterioration. However, the water contact angle decreased for all groups, showing increased wettability. The control group had the lowest contact angle, indicating the highest hydrophilicity.

### 3.4. Surface Roughness

The two-way ANOVA analysis showed that aging had a significant effect (*p* < 0.001) on the surface roughness of the materials. However, the material had no significant influence on the roughness (*p* = 0.22). Additionally, no significant interaction was found between the material and aging (*p* = 0.183). Surface roughness was lowest (0.952 ± 0.044) in the control group at baseline and slightly higher with the addition of 0.5 or 1 wt.% of glass fibers, but the differences among them were non-significant. The surface roughness of all the groups significantly increased after 14 days of aging. The 0.5 wt.% glass fiber group was the smoothest, followed by the 1% glass fiber group. The results are shown in Table 2.

### 3.5. Water Contact Angle

Table 3 presents the two-way ANOVA results of the contact angle data. The material, aging, and the interaction of the material with aging had significant effects (*p* < 0.001) on the contact angle. The control group at baseline had the lowest contact angle, and the 1 wt.% glass fiber group had the highest values. After 14 days, the contact angle was significantly reduced in all the groups, indicating the better wettability of the material over time.

### 3.6. Water Sorption and Solubility

The two-way ANOVA results for water sorption and solubility are presented in Table 4 and Table 5. Aging increased the water sorption of all the groups (*p* < 0.001). The control group had the highest sorption values at both time intervals, whereas the 1 wt.% glass fiber group had the lowest. Both the material and the aging significantly influenced solubility (*p* < 0.001). However, no significant interaction between group and storage time was observed (*p* = 0.988).

## 4. Discussion

This laboratory study investigated the effects of micron-sized glass fiber on the physicomechanical properties of tissue conditioner after 24 h and 14 days of water aging. The study hypothesized that adding glass fiber would improve the tissue conditioner’s physicomechanical characteristics, and these characteristics in the glass fiber-reinforced tissue conditioner would remain unchanged after aging. The hypothesis was partially accepted, as some properties improved by incorporating glass fibers. However, aging had a significant effect on the materials’ characteristics; changes were more prominent in the control group (without glass fibers).

Tensile strength is a crucial characteristic of tissue conditioners that influences their durability and resistance to tearing or deformation during function [23]. Higher tensile strength can indirectly lead to better clinical performance, particularly when combined with an effective adhesive or bonding system [24]. The tensile strength of the control group at 24 h had higher tensile strength than the glass fiber-reinforced groups. This reduction in tensile strength may be attributed to poor interfacial bonding between the glass fibers and the resin matrix. Inadequate fiber-matrix adhesion can lead to stress concentration points, reduced load transfer efficiency, and premature failure under tensile loading [18]. The aging had a significant impact on the strength of the tested materials, causing significant decreases over 14 days, likely due to water absorption, thermal degradation, or oxidative changes that can weaken the polymer network. Although slight differences were noted among groups, with the 0.5 wt.% glass fiber group showing numerically less change in strength, these differences, however, should be interpreted with caution [15].

All groups exhibited shrinkage relative to their baseline dimensions. After 14 days of water immersion, a slight increase in the retained linear dimension was observed compared to the 24-h measurements; however, the samples remained smaller than their original size. The 1 wt.% glass fiber group showed the least change over time, with retained dimensions of 96.07% at 24 h and 96.88% after 14 days. This finding is consistent with previous research documenting the dimensional stability of Visco-Gel™, which typically exhibits shrinkage values between 1% and 3% over time [25].

The water contact angle indicates the material’s hydrophilic and hydrophobic behavior [19]. Tissue conditioners should possess some hydrophilicity to adhere to oral tissues, retain moisture, and stimulate salivary flow to improve patient comfort [23]. However, excessive hydrophilicity can indicate excessive absorption of water, which can reduce its mechanical properties and cause staining/discoloration. Some hydrophobicity is needed for improved flexibility, stability, and durability [26]. Given that, according to ASTM D7334, the contact angle of 45–90° (between hydrophilic and hydrophobic) is appropriate for the material for optimum function. The contact angle of Visco-Gel™ significantly increased after glass fibers incorporation due to the fibers’ hydrophobic nature, and it remained higher than the control after aging, indicating the hydrophobic properties imparted by glass fibers were persistent. However, all groups had contact angle values within the range necessary for optimal performance and longevity after 14 days. Only the 1 wt.% glass fiber group presented a contact angle above 90° (92.204 ± 0.431) after 24 h, which subsequently decreased to within the clinically acceptable range after 14 days (90.114 ± 0.654).

Research studies have shown that tissue conditioners absorb water and release soluble components when exposed to saliva or other liquids. This may break down the polymer network, leading to softening, loss of mechanical integrity, reduced elasticity, and compromised surface structure. These changes can facilitate *C. albicans* colonization and growth, also cause dimensional instability in the material, and reduce the functional efficiency and clinical lifetime of the tissue conditioner [5,9,11]. Ideally, tissue conditioners should have low sorption and solubility for a longer lifetime and effective clinical performance. Visco-Gel™, which was also evaluated by Murata et al., has been reported to exhibit low water solubility (up to 4.0%), consistent with the relatively low values observed in the present study [25]. The addition of glass fibers further reduced these values. Previous studies have shown that the incorporation of additives such as chitosan, nystatin, or chlorhexidine into tissue conditioners tends to increase water sorption and solubility due to their hydrophilic nature [26,27,28]. In contrast, the incorporation of glass fibers in our study led to an increase in water contact angle, indicating a more hydrophobic surface behavior.

Surface roughness is a crucial parameter for assessing the performance of dental biomaterials [29]. Profilometric analysis revealed that all tested groups had surface roughness values above the threshold level of 0.2 μm, which is considered the limit below which microbial adhesion is minimized [30]. The incorporation of glass fibers further increased this surface roughness compared to the control group. At 24 h, the control group exhibited the lowest roughness, and after 14 days, 0.5 wt.% glass fiber group showed the lowest roughness, indicating the ability of its surface to resist bacterial attachment and colonization better over time.

Our current study is the first to reinforce tissue conditioner with glass fibers to improve its physicomechanical properties. The properties of the tested materials were evaluated after aging for 14 days, the usual clinical life of this tissue conditioner. However, our study used one commercial tissue conditioner and performed only 14 days of aging. Long-term aging effects (e.g., thermal and water aging), and dynamic mechanical behavior will provide a more comprehensive understanding of the reinforced material’s performance.

## 5. Conclusions

Incorporating E-glass fibers (150 µm length, 16 µm diameter) reduced the water sorption and solubility of the tissue conditioner, which are favorable properties for successful and effective clinical use. The 0.5 wt.% glass fiber group demonstrated not only the lowest water sorption and solubility values but also exhibited favorable physicomechanical characteristics (surface roughness, tensile strength, dimensional stability, and contact angle). Reinforcement with 0.5 wt.% glass fibers may enhance the material’s suitability for use as a tissue conditioner.

## Figures and Tables

**Table 1:** Descriptive statistics and pair-wise comparison of surface roughness, water sorption and solubility, and contact angle for tested groups.

Aging.	Group	Surface Roughness (µm)	Water Sorption (%)	Water Solubility (%)	Water Contact Angle (°)
		Mean ± SD	Mean ± SD	Mean ± SD	Mean ± SD
Baseline (24 h)	Control	0.95 ± 0.04 ^a^	−2.39 ± 0.50 ^a^	1.43 ± 0.31 ^a^	88.59 ± 0.60 ^ab^
	0.5% wt. GF	0.97 ± 0.05 ^bc^	−2.32 ±0.29 ^bc^	1.39 ± 0.25 ^bce^	90.09 ± 0.92 ^bc^
	1% wt. GF	1.01 ± 0.08 ^b^	−2.25 ± 0.30 ^bde^	1.19 ± 0.11 ^bde^	92.20 ± 0.43 ^abcd^
14 days	Control	1.17 ± 0.06 ^ab^	3.63 ± 0.86 ^ab^	2.05 ± 0.14 ^ab^	71.25 ± 0.89 ^ab^
	0.5% wt. GF	1.01 ± 0.11	2.96± 0.34 ^acd^	2.01 ± 0.18 ^acd^	82.25 ± 0.83 ^abcd^
	1% wt. GF	1.15 ± 0.09 ^ac^	2.78 ± 0.33 ^ace^	1.95 ± 0.16 ^e^	90.11 ± 0.65 ^abd^

Key: Statistical difference between groups is indicated by different superscripts arranged vertically.

**Table 2:** Two-way ANOVA test result for surface roughness.

Parameters	Sum of Squares	df	Mean Square	f-Value	*p*-Value
Material	0.0192	2	0.01	6.95	0.22
Aging	0.168	1	0.168	27.874	<0.001
Material*Aging	0.022	2	0.011	1.826	0.183
Error	0.145	24	0.006		

Key: * = Interaction between Material and Aging.

**Table 3:** Two-way ANOVA test result for water contact angle.

Parameters	Sum of Squares	df	Mean Square	f-Value	*p*-Value
Material	634.359	2	317.18	574.974	<0.001
Aging	619.983	1	619.983	1123.888	<0.001
Material*Aging	296.81	2	148.405	269.204	<0.001
Error	13.239	24	0.552		

Key: * = Interaction between Material and Aging.

**Table 4:** Two-way ANOVA test result for water sorption.

Parameters	Sum of Squares	df	Mean Square	f-Value	*p*-Value
Material	0.731	2	0.366	1.567	0.229
Aging	222.06	1	222.06	951.64	<0.001
Material*Aging	1.314	2	0.657	2.816	0.080
Error	5.599	24	0.233		

Key: * = Interaction between Material and Aging.

**Table 5:** Two-way ANOVA test result for water solubility.

Parameters	Sum of Squares	df	Mean Square	f-Value	*p*-Value
Groups	0.375	2	0.188	5.257	0.013
Aging	2.803	1	2.803	78.58	<0.001
Groups*Aging	0.001	2	0	0.012	0.988
Error	0.856	24	0.036		

Key: * = Interaction between Material and Aging.

## Data Availability

The data presented in this study are available on reasonable request from the corresponding author.

## References

[1] Mohammed G.A. (2023). Studying the Anti Candidal-Activity of Different Herbal Oils Incorporated into Tissue Conditioner: (A Comparative study). Jordan J. Pharm. Sci..

[2] Homsiang W., Kamonkhantikul K., Arksornnukit M., Takahashi H. (2021). Effect of zinc oxide nanoparticles incorporated into tissue conditioner on antifungal, physical, and mechanical properties. Dent. Mater. J..

[3] Iqbal Z., Zafar M.S. (2016). Role of antifungal medicaments added to tissue conditioners: A systematic review. J. Prosthodont. Res..

[4] Sharma S., Hegde V. (2014). Comparative evaluation of antifungal activity of melaleuca oil and fluconazole when incorporated in tissue conditioner: An in vitro study. J. Prosthodont..

[5] Krishnamoorthy G., Narayana A.I., Peralam P.Y., Balkrishanan D. (2019). To study the effect of Cocos nucifera oil when incorporated into tissue conditioner on its tensile strength and antifungal activity: An in vitro study. J. Indian Prosthodont. Soc..

[6] Lee H.-L., Wang R.-S., Hsu Y.-C., Chuang C.-C., Chan H.-R., Chiu H.-C., Wang Y.-B., Chen K.-Y., Fu E. (2018). Antifungal effect of tissue conditioners containing poly (acryloyloxyethyltrimethyl ammonium chloride)-grafted chitosan on *Candida albicans* growth in vitro. J. Dent. Sci..

[7] Ganokwalai N., Chotprasert N., Choonharuangdej S., Shrestha B., Srithavaj T. (2024). Mechanical properties of dental tissue conditioner containing lemongrass essential oil. J. Prosthet. Dent..

[8] Kitagawa Y., Yoshida K., Takase K., Valanezhad A., Watanabe I., Kojio K., Murata H. (2020). Evaluation of viscoelastic properties, hardness, and glass transition temperature of soft denture liners and tissue conditioner. Odontology.

[9] Mikulewicz M., Chojnacka K., Raszewski Z. (2023). Comparison of mechanical properties of three tissue conditioners: An evaluation in vitro study. Medicina.

[10] Hashem M.I. (2015). Advances in soft denture liners: An update. J. Contemp. Dent. Pract..

[11] Neppelenbroek K.H. (2016). Sustained drug-delivery system: A promising therapy for denture stomatitis?. J. Appl. Oral Sci..

[12] Nam K.Y., Lee C.J. (2019). Characterization of silver nanoparticles incorporated acrylic-based tissue conditioner with antimicrobial effect and cytocompatibility. Nanobiomaterials in Clinical Dentistry.

[13] Khan A.A., Zafar M.S., Fareed M.A., AlMufareh N.A., Alshehri F., AlSunbul H., Lassila L., Garoushi S., Vallittu P.K. (2023). Fiber-reinforced composites in dentistry—An insight into adhesion aspects of the material and the restored tooth construct. Dent. Mater..

[14] Väkiparta M., Koskinen M., Vallittu P., Närhi T., Yli-Urpo A. (2004). In vitro cytotoxicity of E-glass fiber weave preimpregnated with novel biopolymer. J. Mater. Sci. Mater. Med..

[15] Matinlinna J.P., Lung C.Y.K., Tsoi J.K.H. (2018). Silane adhesion mechanism in dental applications and surface treatments: A review. Dent. Mater..

[16] Hatamleh M.M., Maryan C.J., Silikas N., Watts D.C. (2010). Effect of net fiber reinforcement surface treatment on soft denture liner retention and longevity. J. Prosthodont. Implant Esthet. Reconstr. Dent..

[17] Singh K., Sharma S.K., Negi P., Kumar M., Rajpurohit D., Khobre P. (2016). Comparative evaluation of flexural strength of heat polymerised denture base resins after reinforcement with glass fibres and nylon fibres: An in vitro study. Adv. Hum. Biol..

[18] Gad M.M., Fouda S.M., Al-Harbi F.A., Näpänkangas R., Raustia A. (2017). PMMA denture base material enhancement: A review of fiber, filler, and nanofiller addition. Int. J. Nanomed..

[19] Alsunbul H., Khan A.A., Alqahtani Y.M., Hassan S.A.b., Asiri W., Saadaldin S., Alharthi R., Aldegheishem A. (2023). Using functionalized micron-sized glass fibres for the synergistic effect of glass ionomer on luting material. J. Funct. Biomater..

[20] (2016). Dentistry—Soft Lining Materials for Removable Dentures—Part 2: Materials for Long-Term Use.

[21] (2018). Dentistry—Soft Lining Materials for Removable Dentures—Part 1: Materials for Short-Term Use.

[22] Chladek G., Żmudzki J., Kasperski J. (2014). Long-term soft denture lining materials. Materials.

[23] Prasad A., Prasad B.R., Shetty V., Shastry C. (2014). Tissue conditioners: A review. J. Health Allied Sci. NU.

[24] Khan A.A., Bari A., Abdullah Al-Kheraif A., Alsunbul H., Alhaidry H., Alharthi R., Aldegheishem A. (2023). Oxidized natural biopolymer for enhanced surface, physical and mechanical properties of glass ionomer luting cement. Polymers.

[25] Murata H., Kawamura M., Hamada T., Saleh S., Kresnoadi U., Toki K. (2001). Dimensional stability and weight changes of tissue conditioners. J. Oral Rehabil..

[26] Lima J.F., Maciel J.G., Arrais C.A., Porto V.C., Urban V.M., Neppelenbroek K.H. (2016). Effect of incorporating antifungals on the water sorption and solubility of interim resilient liners for denture base relining. J. Prosthet. Dent..

[27] Saeed A., Zahid S., Sajid M., Ud Din S., Alam M.K., Chaudhary F.A., Kaleem M., Alswairki H.J., Abutayyem H. (2022). Physico-mechanical properties of commercially available tissue conditioner modified with synthesized chitosan oligosaccharide. Polymers.

[28] Urban V.M., De Souza R.F., Galvao Arrais C.A., Borsato K.T., Vaz L.G. (2006). Effect of the association of nystatin with a tissue conditioner on its ultimate tensile strength. J. Prosthodont..

[29] Khan A.A., De Vera M.A.T., Mohamed B.A., Javed R., Al-Kheraif A.A. (2022). Enhancing the physical properties of acrylic resilient denture liner using graphene oxide nanosheets. J. Vinyl Addit. Technol..

[30] Khan A.A., Al-Khureif A.A., Al-Mutairi M., Al-Majed I., Aftab S. (2024). Physical and mechanical characterizations of experimental pit and fissure sealants based on bioactive glasses. J. Clin. Pediatr. Dent..

